# Assessment of Physical, Mechanical, Biopharmaceutical Properties of Emulgels and Bigel Containing Ciclopirox Olamine

**DOI:** 10.3390/polym14142783

**Published:** 2022-07-07

**Authors:** Agnė Mazurkevičiūtė, Inga Matulytė, Marija Ivaškienė, Modestas Žilius

**Affiliations:** 1 Institute of Pharmaceutical Technologies, Lithuanian University of Health Sciences, 50162 Kaunas, Lithuania; inga.matulyte@lsmu.lt (I.M.); modestas.zilius@lsmu.lt (M.Ž.); 2 Department of Clinical Pharmacy, Lithuanian University of Health Sciences, 50162 Kaunas, Lithuania; 3 Department of Drug Technology and Social Pharmacy, Lithuanian University of Health Sciences, 50162 Kaunas, Lithuania; 4 Dr. L. Kriauceliunas Small Animal Clinic, Veterinary Academy, Lithuanian University of Health Sciences, 47181 Kaunas, Lithuania; marija.ivaskiene@lsmu.lt

**Keywords:** emulgel, bigel, oleogel, oil, ciclopirox olamine, poloxamer 407, antifungal, rheology, texture analysis

## Abstract

Emulsions are thermodynamically unstable systems and it is difficult to produce biphasic formulations with large amounts of oil. The aim of our study was to prepare biphasic formulations containing 1% ciclopirox olamine and to determine the influence of the method of oil incorporation (without and with emulsifier and gelifier) on the physical (pH, particle size, rheological properties), mechanical, and biopharmaceutical properties of the formulations. It was found that the use of a poloxamer 407 gel as the hydrophase could result in a stable formulation when an oil with (EPG) or without an emulsifier (APG) or oleogel (OPG) was used as the oily phase. The results of the studies showed that the addition of an emulsifier (polysorbate 80) led to a decrease in the sol-gel temperature, a slower release of ciclopirox olamine, and a higher stability in the freeze–thaw test. However, regardless of the way the oil is incorporated, the particles are distributed in the same range and the antifungal activity against *T. rubrum* is the same. It is possible to create a biphasic formulation with a large amount of oil and poloxamer gel, but for greater stability, it is recommended to include an emulsifier in the composition.

## 1. Introduction

Emulsions are dispersion systems that consist of two immiscible liquids (water and oil) and an emulsifying agent. The dispersed phase forms particles that are surrounded by continuous phases. These dispersion systems can be of two types: oil-in-water (O/W) and water-in-oil (W/O) emulsions [1,2] or multiple emulsions types (O/W/O and W/O/W) [3]. The stability of emulsions is affected by two groups of factors: emulsion components and preparation technology [4]. In order to obtain the highest quality final product, it is important to achieve the proper physical properties (particle size, flow behavior, and physical stability) of the emulsions [1]. The emulsifiers and stabilizing agents (various surfactants, polymers) are necessary to increase their long-term stability because emulsions are thermodynamically unstable systems [1]. The emulsion instability often occurs as flocculation, coalescence, creaming, and Ostwald ripening [3,5]. In this way, the emulsions separate into two phases over time. The shelf life and quality of emulsions are highly dependent on particle size and aggregation. The increase in particle size is one of the main reasons for the loss of stability [3,6]. The stability of emulsions is greatly influenced by temperature and pH [5]. The interfacial film formed by molecules of surfactant is a very important factor in controlling the stability of the emulsion [4]. These surface-active molecules reduce surface tension and prevent particle flocculation [6] or coalescence [2]. Costa et al. confirmed that cellulose as an amphiphilic biopolymer increases viscosity and forms a 3D interconnected network that detains the oil particles by limiting their dynamics and improving emulsion stability [1]. Kori et al. revealed that carboxyl methyl cellulose (CMC) increased the stability of the emulsion, as it is used as a thickener to increase the viscosity of the aqueous continuous phase and thus inhibits creaming [3]. One of the main challenges is to produce kinetically stable emulsions for a certain period to increase the shelf life [3].

Emulgels are formed by mixing emulsions with gels containing gelling agents. Emulgels have the ability to deliver hydrophilic and lipophilic drugs. These semi-solid systems not only have the advantages of emulsions and gels but are also stable [7,8]. O/W emulgels or O/W emulsion hydrogels can deliver lipid-soluble compounds and have good physical stability and mechanical properties as gels [9,10]. Emulgels have an acceptable and pleasant appearance, convenient spreadability, emollient effect, controlled viscosity, thixotropic and ecological properties, have a higher ability to penetrate into the skin, and a longer shelf life [11].

Bigels are biphasic systems consisting of two gelled phases [12]. They are of three types: O/W (an organogel dispersed into hydrogel), W/O (a hydrogel dispersed into organogel), or a bi-continuous system [13]. Pharmaceutical and cosmetic bigels focus on the ability to deliver both hydrophilic and lipophilic compounds, good spreadability, water washability, and moisturizing and emollient effects [14,15]. The structure and distribution of each phase play an important role in the development of the biphasic systems [14].

Poloxamer 407 is triblock copolymer, formed by one poly-[propylene oxide] (POO) block and two blocks of poly-[ethylene oxide] (PEO): HO[CH_2_-CH_2_O]_95-105_[CH(CH_3_)-CH_2_O]_54-60_[CH_2_-CH_2_]_95-105_OH [16]. Micelles with hydrophobic interiors and hydrophilic mantles are formed [17]. Critical micellar temperature depends on concentration and other substances [18].

Due to the unique properties of poloxamer 407, it is suitable for the production of biphasic semi-solid formulations: emulgels and bigels [19]. Grela et al. revealed that up to 60–85% oil (depending on the oil) can be added to 17% poloxamer gel [20]. Ferreira and Brushi revealed that stable emulgels with a poloxamer are obtained at a poloxamer concentration of 15–20%. In this case, the sol-gel transition temperature is between 25 °C and 27 °C. Our goal is to produce stable formulations that are rigid at room temperature (i.e., the sol-gel transition temperature would be lower than 20 °C). Therefore, we chose to use 25% poloxamer gel for the production.

Additional excipients may be used in the manufacture of emulgels with a poloxamer: surfactants and bioadhesive polymer [19,21,22]. Polysorbate 80 (polyoxyethylene sorbitan monooleate) was chosen as the emulsifier. Polysorbate molecules intervene between poloxamer molecules in the micelle shell [17]. It is known that the effect of polysorbate is enhanced by amphiphilic substances, resulting in stronger micelles than poloxamer alone due to the synergistic effect [23]. Fumed silica (SiO_2_) was chosen to gel the oil. Fumed silica formed branched agglomerates and it maintain oleogel structure [24,25].

The release of the active ingredients from the pharmaceutical form and the penetration into the skin are influenced by the properties of the molecule: size, lipophilicity/hydrophilicity, pKa value, water solubility, and others. Different solutions are required to ensure sufficient release of the active substances with different properties from the pharmaceutical form and penetration into the skin. We selected the antifungal active substance ciclopirox olamine for this study. Ciclopirox olamine is a synthetic, broad-spectrum antifungal substance whose molecular weight is 268.35 Da, logP is 2.15, sparingly soluble in water (14.41 mg/mL), and ciclopirox pKa is 6.84 [26]. The minimum inhibitory concentration of ciclopirox olamine for most human pathogenic fungi is 0.5–3.9 µg/mL [27].

Emulgels are a promising pharmaceutical form for poorly water-soluble substances. The oil in the formulations softens the skin and prevents significant water loss through the skin. Formulations with low water content are greasy and unpleasant to use, but high oil content in formulations often results in instability. In this work, we aimed to produce stable formulations with a high (50 percent) oil content. Although other researchers have already produced emulgels with a poloxamer, there is a lack of scientific data on the influence of oil administration methods on the properties and stability of emulgels with poloxamer 407. Therefore, in this work we added the oil in three different ways: without and with emulsifier (emulgels) and gelifier (bigel).

The aim of this study was to evaluate the influence of the methods of oil incorporation on the physical, mechanical, biopharmaceutical properties, and antifungal activity of emulgels and bigel.

## 2. Materials and Methods

### 2.1. Materials

Mineral oil, Poloxamer 407 (Kolliphor^®^ P 407) (Oxyethylene 71.5–74.9%) were received from Sigma-Aldrich (St. Louis, MO, USA). Fumed silica (Aerosil 300) (specific surface area 270–330 m^2^/g, SiO_2_ content > 99.8%) was purchased from Evonik Industries (Hanau, Germany). Ciclopirox olamine (>98%) was supplied by Chemical Point (Oberhaching, Germany).

For ultra-performance liquid chromatography (UPLC) analysis, acetonitrile (≥99.9%) and trifluoracetic acid (99%) were purchased from Sigma Aldrich (St. Louis, MO, USA).

Water was purified by Thermo Scientific Pacific RO 7 (Niederelbert, Germany). Water for chromatography was processed with GenPure Pro UV-TOC from Thermo Scientific (Langenselbold, Germany) to obtain water with a resistivity of up to 18.3 MΩcm at 25 °C.

### 2.2. Methods

#### 2.2.1. Production of the Experimental Semi-Solid Formulations

**Hydrogel**. The hydrogel was prepared by mixing poloxamer 407 (25%) with purified water at 500 rpm using a magnetic stirrer at 5 °C until poloxamer 407 was fully dissolved. The drug-loaded hydrogel was prepared by dispersing ciclopirox olamine in poloxamer 407 solution so as to have a final concentration of 1% (*w*/*w*) in the formulations. Hydrogels were kept at room temperature (20 ± 2 °C) overnight.

**Oleogel**. For the preparation of the oleogel, gelling agent (fumed silica, 5% *w*/*w*) was gradually added to mineral oil and stirred at 500 rpm using the magnetic stirrer (IKAMAG^®^ C-MAG HS-7 (IKA-Werke GmbH & Co.KG, Staufen, Germany) at 80 °C.

**Emulgel** (APG). Mineral oil was mixed with hydrogel in a ratio of 1:1 using a mixer Unguator.

**Emulgel** (EPG). Mineral oil with polysorbate 80 was mixed with hydrogel in a ratio of 1:1 using a mixer Unguator.

**Bigel** (OPG). Oleogel was mixed with hydrogel in a ratio of 1:1 using a mixer Unguator.

The final compositions of the experimental semi-solid formulations are given in Table 1.

#### 2.2.2. Particle Size and Distribution Measurements

Oil particle size and distribution were assessed using Mastersizer 3000 with Hydro EV unit (Malvern Panalytical Ltd., Malvern, UK). Samples without dilution were added dropwise in the dispersant (water) to obtain laser obscuration between 9.5% and 10.5%. The pump speed was kept constant at 2400 rpm. The refractive index used for dispersant and dispersing material were 1.330 and 1.467 respectively. Particle size distribution was measured in five runs and the average was calculated. The formulations were described by the percentiles (D10, D50, and D90) values.

#### 2.2.3. Evaluation of Sol-Gel Transition Temperature

The analysis of gelation temperature sol-gel was performed using rotational rheometer MCR102 (Anton Paar BmbH, Graz, Austria) equipped with a plate–plate geometry (gap 1.0 mm); the temperature was controlled with a Peltier system. The temperature ramp analysis was performed over the range from 40 to 0 °C within the linear viscoelastic region conditions (angular frequency 10 rad/s, deformation 0.2%). The rate of heating was 1 °C/min. The storage modulus (G′) and loss module (G″) were calculated. Tsol/gel was considered the temperature at which G′ was halfway between solution and gel.

#### 2.2.4. Texture Analysis of Semi-Solid Formulations

Texture analysis was performed using TA.XT Plus Texture Analyzer (Stable Micro Systems, Godalming, UK). Firmness, consistency, cohesiveness, and index of viscosity were measured. All these parameters were measured using the back extrusion method for semi-solid products. Parameters: pre-test speed 1.5 mm/s, test speed 2 mm/s, post-test speed 2 mm/s, distance 10 mm, trigger force 0.294 N (30 g).

The gel was placed in a special container and the test was run according to the set parameters. The samples were analyzed in triplicate, and the average values were calculated.

#### 2.2.5. pH Measurement

The experimental semi-solid formulations pH was measured using a pH meter (766 Callimatic (Knick, Berlin, Germany)) with an electrode (SE 104 N).

#### 2.2.6. Centrifugation of Semi-Solid Formulations

Safe-lock tubes (2.0 mL) (Eppendorf, Hamburg, Germany) were used for centrifugation test; 1.7 ± 0.1 g of the test formulation was added to the tubes.

The initial stability of the experimental semi-solid systems was assessed by centrifugation (10,000 rpm, 10 min) (Thermo Scientific Biofuge Stratos centrifuge, Osterode, Germany). The system was considered stable if oil separation was not observed after centrifugation.

#### 2.2.7. Stability Test

Two types of stability studies were performed: freeze–thaw and cooling–heating.

1. The freeze–thaw test consisted of 12 h of freezing (−20 ± 1 °C) followed by 12 h of thawing (20 ± 1 °C). Freeze–thaw cycles were repeated 5 times.
2. The cooling–heating test consisted of 12 h of cooling (5 ± 1 °C) followed by 12 h of heating (40 ± 1 °C). Cooling–heating cycles were repeated 5 times.

**Figure 1:**
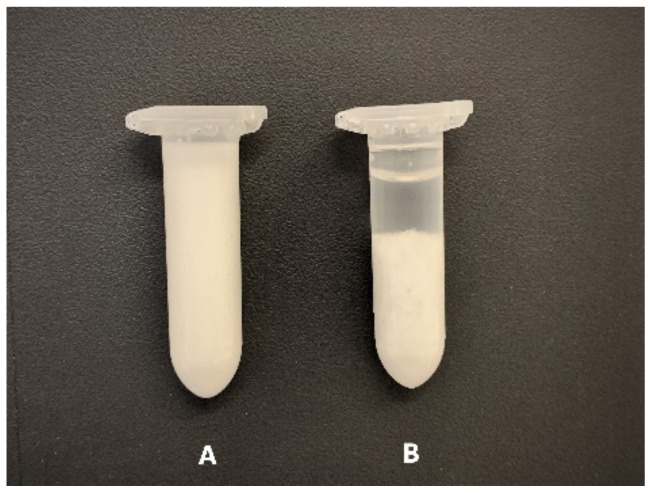
Example of formulations after stability test: A—stable formulation, no visible physical changes; B—unstable formulation with phase separation.

Safe-lock tubes (2.0 mL) (Eppendorf, Hamburg, Germany) were used for stability tests; 1.7 ± 0.1 g of the test formulation was added to the tubes. At the end of each cycle, the formulations were checked for any visible, physical changes. Particle size was measured after each cycle. When physical phase separation was observed (example in Figure 1), the formulation was no longer investigated.

#### 2.2.8. In Vitro Release Test

An in vitro release test was performed with flow-through cells (Sotax CE7 smart with pump). Cellulose membrane (Sigma Aldrich (St. Louis, USA)) was mounted in adapters for semi-solid formulations. The donor phase (1 ± 0.1 g) was placed into the adapter. The diffusion area was 1.33 cm^2^. The aqueous receptor medium was 50 mL of purified water. Selected conditions fulfilled the criteria of sink conditions. Samples of the solution were collected at 1, 2, 3, 4, 5, and 6 h and replaced with the same volume of fresh water.

All samples were analyzed by liquid chromatography using the Acquity UPLC H-Class chromatography system (Waters, Milford, MA, USA) equipped with DAD (Waters, Milford, MA, USA), performing detection at 303 nm. Validated UPLC method conditions were C18 column (130 Å, 1.7 µm, 2.1 mm × 50 mm, Waters, Milford, MA, USA), solvent A (acetonitrile) and solvent B 0.5% (*v*/*v*) of trifluoracetic acid in ultrapure water) ratio 40–60%; the injection volume was 5 μL; the flow rate was 0.7 mL/min; the column temperature was 25 °C. A standard calibration curve was built up by using standard solutions (4–324 µg/mL). All samples were filtered using a polyvinylidene difluoride filter (pore size 0.2 µm).

The cumulative amounts of released ciclopirox olamine expressed per unit area (cm^2^) were calculated and discussed in the results section.

#### 2.2.9. Antifungal Activity Study

The inhibitory activity of the emulgels and bigel against zoonotic dermatophyte *Microsporum canis* was assessed by the agar well diffusion method. Five strains of *M. canis* isolated from clinical samples of cats were evaluated. The fungi suspension was prepared following the National Committee for Clinical Laboratory Standard protocol (NCCLS, 2002). The agar well diffusion test was performed using potato dextrose agar. The agar plate surface was inoculated by spreading 10 µL of the fungal inoculum over the entire agar surface. The inoculum used was prepared using the *M. canis* from a 5-day culture on Sabouraud dextrose agar, and a suspension was created in a sterile saline solution. The turbidity of the suspension was adjusted with a spectrophotometer at 530 nm to obtain a final concentration to match that of a 0.5 McFarland standard (1–5 × 10^6^). A hole with a diameter of 8 mm was punched aseptically with a sterile metal pipe, and a volume of 50 µL of the formulations was introduced into the well. Each pharmaceutical form was placed into a separate plate. Then, agar plates were incubated at 29 °C for 5 days to allow for fungal growth. Inhibition zone diameters were measured in millimeters. Reading of inhibition diameter zones (mm) was performed at 72 h. Zone diameters were measured to the nearest whole millimeter at a point at which there was no visible growth (100% inhibition).

#### 2.2.10. Statistical Analysis

Statistical analysis of the results was performed by calculating the means (standard deviations) of the measurements of three independent experiments using the IBM SPSS statistics 27 software. Non-parametric tests were used to investigate the differences between the characteristics of the formulations (Mann–Whitney U test and Wilcoxon test for 2 variables and Kruskal–Wallis test and Friedman test for 3 and more variables). The differences were statistically significant at *p* < 0.05.

## 3. Results

### 3.1. Particle Size and Distribution Measurements

The particle size and their distribution are important parameters for all dispersions. Dispersions with smaller particle sizes are generally more stable than with large particles [28].

**Figure 2:**
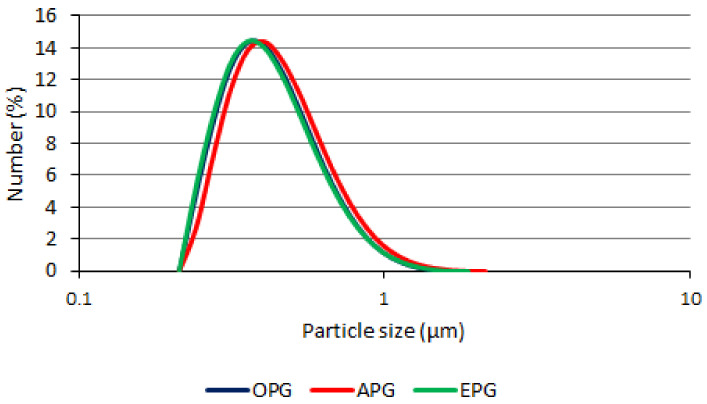
Particle size distribution.

Figure 2 shows particle size distribution of emulgels and bigel. The method of oil application was found to have a negligible effect on particle sizes and distribution, as the particles of all tested formulations were found to be distributed in the same range: OPG 0.214–1.88 µm, APG 0.214–2.13 µm, EPG 0.214–1.88 µm.

The percentiles D10, D50, and D90 indicate the size for which 10, 50, and 90 percent of the particles are equal to or less, respectively. The mean diameter D50 is such a distribution that 50% of the particles have a volume equal to or less. No statistically significant differences were found among the different formulations comparing the percentiles D10, D50, and D90 (*p* = 0.06, *p* = 0.67, *p* = 0.99, respectively).

Particle size was measured immediately after preparation and at 1, 2, 3, and 4 weeks (Table 2, Table 3 and Table 4).

Throughout the study, the OPG systems D10, D50, D90 were 0.283–0.337 µm, 0.420–0.422 µm, and 0.700–0.708 µm, respectively. The APG systems D10, D50, D90 were 0.327–0.371 µm, 0.484–0.567 µm, and 0.865–0.992 µm, respectively. Meanwhile, the EPG systems D10, D50, D90 were 0.275–0.297 µm, 0.404–0.470 µm, and 0.681–0.744 µm, respectively.

Immediately after formulation in the case of D10, the EPG particles were smaller than the OPG and APG particles. After 4 weeks, OPG and EPG particles were smaller than APG. Immediately after production, in the case of D50, OPG particles were smaller than APG and EPG. After 4 weeks, OPG and EPG particles were smaller than APG. Both after production and after 4 weeks in the case of D90, OPG and EPG particles were smaller than APG. However, in both cases (after production and after 4 weeks), the differences were not statistically significant.

It was observed that although APG particles increased during the test in all cases (D10, D50, D90), the increase after 4 weeks was not statistically significant. The changes of the particle size in OPG and EPG were less pronounced and not statistically significant. This may be due to the biphasic structure reinforced by the emulsifier or organogelator. Similar results were obtained with carbopol 940 emulgels [29].

### 3.2. Evaluation of Sol-Gel Transition Temperature

The tested formulations consisted of a poloxamer gel and an oily phase. Poloxamer has unique properties: at low temperatures, poloxamer solutions are liquid, and as the temperature increases, the viscosity of the poloxamer 407 solution increases, and a stiff gel is formed [27]. The sol-gel temperature is the temperature at which the formulation transitions from a rigid to a liquid (or vice versa) state. Knowing the sol-gel temperature, we can predict the behavior of the formulation at different temperatures and select the most suitable production and storage conditions. The aim of this test was to determine the sol-gel transition temperature.

**Figure 3:**
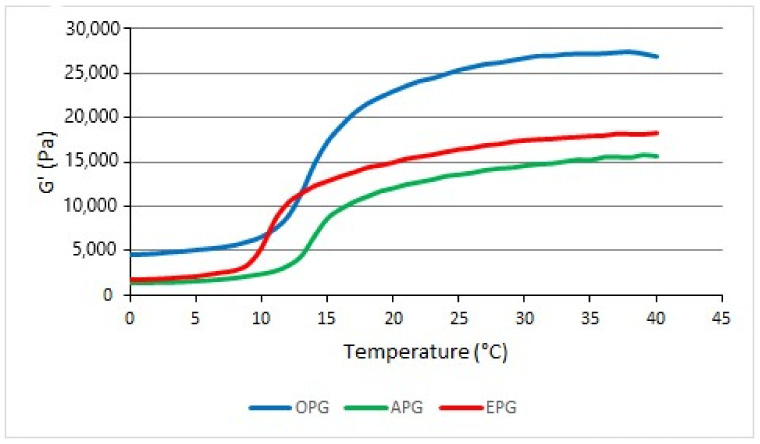
Rheogram of bigel and emulgels.

The behavior of the tested formulations was found to be the same as poloxamer hydrogel: the viscosity was low at low temperatures and the viscosity increased with increasing temperature. The storage modulus (G′) dependence of the temperature is shown in Figure 3. Sol-gel temperature was considered the temperature at which G′ was halfway between solution and gel. Sol-gel transition temperature was determined as OPG 14 °C, APG 14.5 °C, and EPG 10 °C. The values of the loss module (G″) increased and then decreased. The location of the peak coincidesd with the sol-gel temperatures determined from the change in G′. G″ values remained lower than G′ throughout the study.

The addition of polysorbate 80 (EPG) reduced the gelation temperature and formed a gel at a lower temperature than in formulations without polysorbate 80. Polysorbate 80 interferes with the micelles formed by the poloxamer and enhances the structures formed by the poloxamer. Fumed silica increases the viscosity but does not affect the gelation temperature. All formulations had a rigid consistency at room temperature and became liquid when stored in a refrigerator (5 ± 1 °C). The viscosity of formulations applied to the skin was slightly increased, so the formulations did not run off from the site of application.

By measuring the gelation temperature of emulgels poloxamer, it was determined that the gelation temperature decreased with increasing poloxamer 407 concentration [21,30]. In another study [30], 25% poloxamer 407 gel with 50% oil did not form a stiff gel, though in our study stiff emulgels were formed. These differences may be due to the different excipients used.

### 3.3. Texture Analysis of Semi-Solid Formulations

Firmness indicates to some extent the ease with which a semi-solid product can be spread, and it is important to know that batches made at different times have the same spreadability. Products with a low consistency can be more thoroughly applied on the skin and more easily absorbed. On the other hand, a product with a high consistency can be used to cover a wound effectively. A product’s viscosity is defined by its resistance to flow and in skincare, the higher the viscosity, the thicker the substance. The cohesiveness parameter shows the simulating force required to extrude the sample and describes the value of deformation before rupture [31,32,33].

After comparing firmness of all products, it was determined that all samples had higher than 1500 g firmness, they were viscous and easy to apply, and the emulgels and bigel did not run off from the application area. The highest firmness was OPG sample, and the lowest was APG (results are shown in Table 5). Statistically significant differences of firmness were determined in APG and OPG samples (*p* = 0.034). Bigel containing 3% of oleogel gelificator had 1284.84 g of firmness and 6% had 1218.56 g (oleogel and hydrogel ratio was 1:99) [34]. In this study, the oleogel gelicifator amount was 2.5%. After comparing hydrogel gelificator amount 2% of sodium alginate and 12.5% of polaxamer 407, it was determined that APG emulgel had 1.27 times higher firmness. The bigel (OPG) had the highest consistency (8257.48 ± 492.92 g). This parameter shows the thickness and viscosity of the product, the higher the parameter is, the higher these properties are, and vice versa [15]. In addition, statistically significant results were found between OPG and APG samples (*p* = 0.034, parameters of consistency and index of viscosity). The difference between values of cohesiveness parameter was not statistically significant (*p* > 0.05) in all samples. All four parameters of texture evaluated by back extrusion method were highest in the OPG sample (cohesiveness and index of viscosity meanings were negative but their value was highest), middle in EPG, and lowest in APG. These results show that bigel (gel with poloxamer 407 mixed with silicon dioxide gel) creates the strongest structure of the sample. Polysorbate 80 as an excipient increased firmness and other parameters in EPG sample, but not statistically significantly compared with the APG sample.

**Figure 4:**
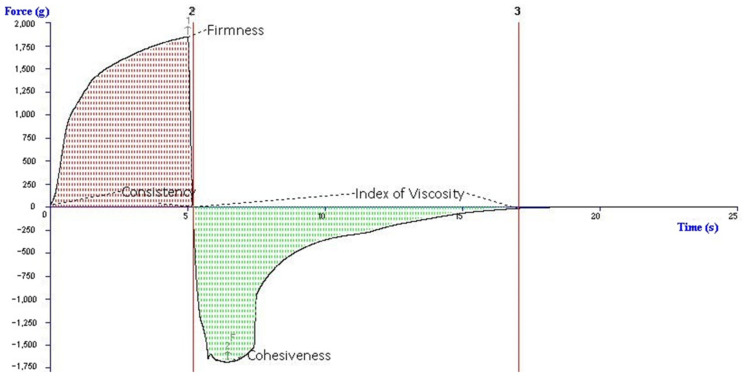
Representative graph of texture parameters by back extrusion test.

The texture analysis representative graph with all areas of parameters (firmness, consistency, cohesiveness, and index of viscosity) is presented in Figure 4.

### 3.4. pH Measurement

Emulgel APG pH was 9.3 (0.1), emulgel EPG pH was 9.2 (0.1), and bigel OPG pH was 9.0 (0.1). These results show that the experimental semi-solid formulations are alkaline probably due to ciclopirox olamine because the excipients are neutral, nonionic substances (mineral oil, poloxamer, polysorbate, silicon dioxide). The pH of the skin surface was 4–6. Due to the alkaline pH, the formulations should not be applied to skin as it may be irritating to it. It is therefore necessary to adjust the pH of these semi-solid formulations.

### 3.5. Centrifugation of Semi-Solid Formulations

One of the quality tests for emulsion systems is centrifugation. Centrifugation of less stable formulations separates the phases. The formulations remained stable while they were undergoing the process of centrifugation at 10,000 rpm for 10 min. This indicates that the poloxamer 407 forms a solid structure gel and is capable of maintaining an emulsion system regardless of the method of oil incorporation: by incorporation of pure oil, by the use of emulsifiers in oil, or by gelation of the oil.

### 3.6. Stability Test

Emulgels with poloxamer and high oil content are less stable at low temperatures due to increasing particles [21]. Therefore, it is important to determine the stability of the produced emulgels and bigels at low temperatures.

Poloxamer 407 has unique properties: the gels of poloxamer 407 liquefy upon cooling, and there is a risk that the phases of the emulsion systems with poloxamer 407 will separate. Two types of cold stability tests were performed: cooling–heating test and freeze–thaw test.

**Figure 5:**
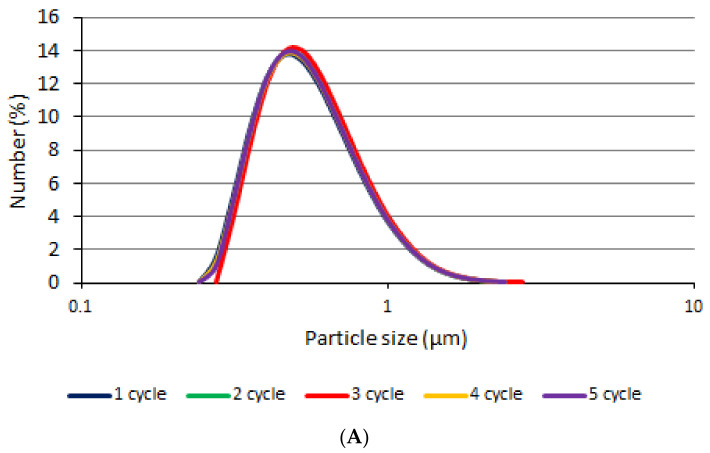

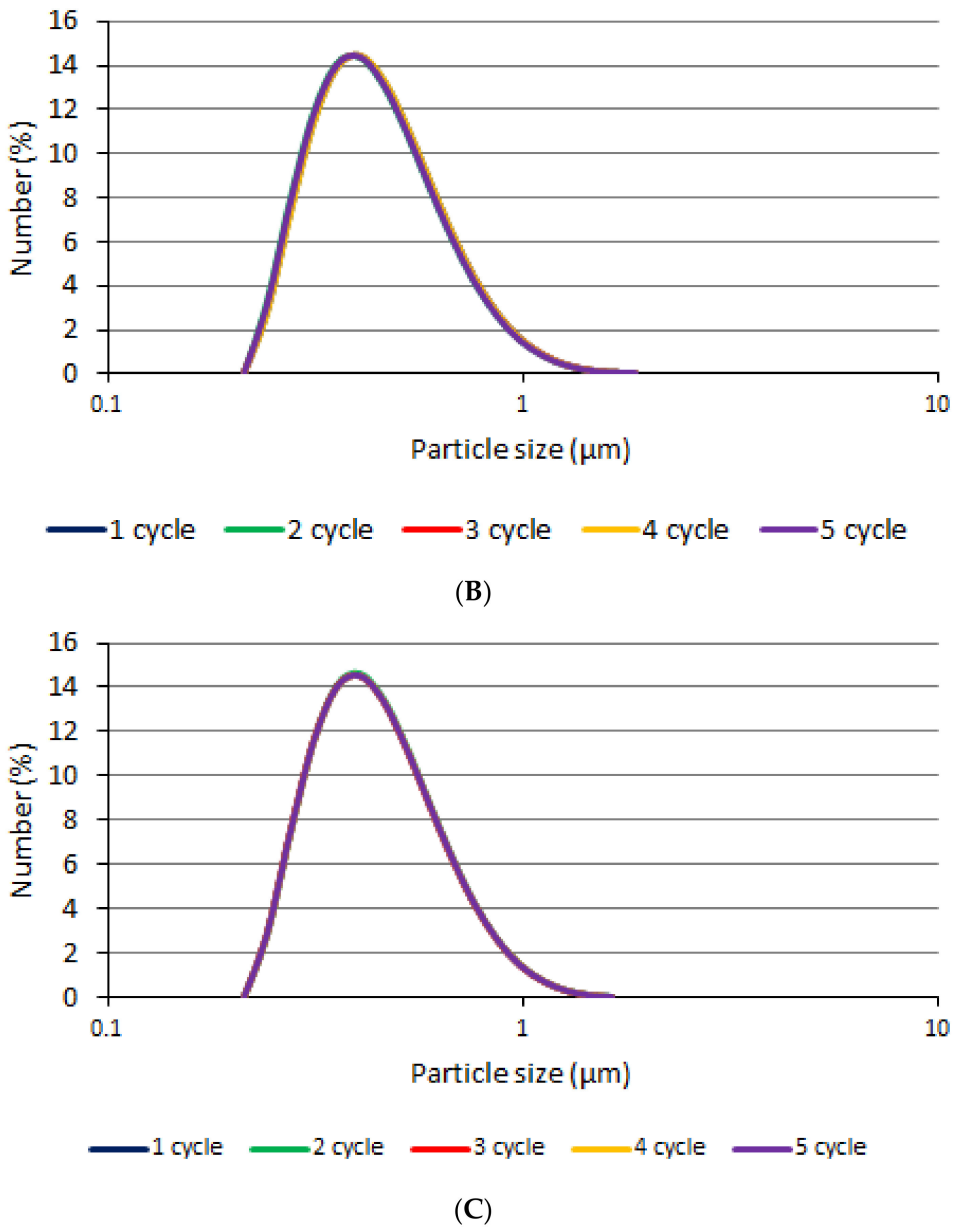
The particle size during the cooling–heating test: (**A**)—APG, (**B**)—EPG, (**C**)—OPG.

The cooling–heating test shows the stability of the formulations when the temperature decreases below the sol-gel temperature and returns again. No visual changes were observed throughout the study. After measuring the particles after each cycle, it was found that the particles were found not to change during the study and to remain in the same range. The histograms are shown in Figure 5.

The freeze–thaw test shows the stability of formulations when formulations not only liquefy but also freeze. The formation of ice crystals can disrupt the emulsion structure, so it is important to determine the freezing behavior of formulations [21].

**Figure 6:**
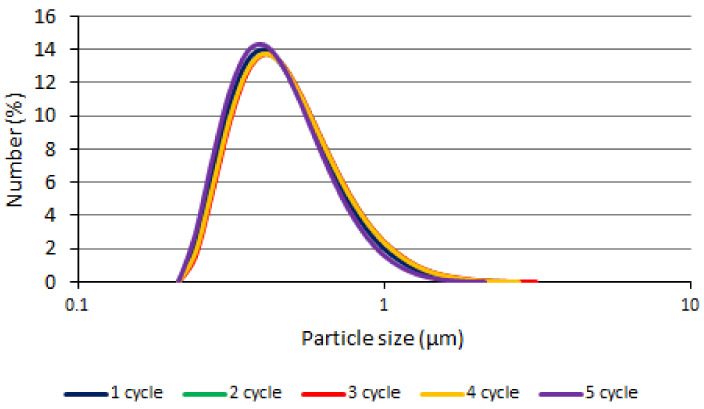
The particle size of EPG during the freeze–thaw test.

In the freeze–thaw test, the phases of APG and OPG were separated after the first cycle. No changes in EPG were observed visually throughout the study (over five cycles). Particle size measurements (Figure 6) have shown that EPG particles remained in the same range throughout the study. Percentile D10 was within range 0.312–0.327 µm, D50 0.461–0.493 µm; D90 0.781–0.872 µm. No statistically significant increase in particles was observed, so it can be argued that the addition of an emulsifier strengthens the emulsion system.

The temperature in the refrigerator remained high enough for the poloxamer molecules to retain the micelles, so all formulations remained stable. The temperature in the freezer (−20 °C) is lower than the micelle formation temperature, so phase separation was observed in formulations in which the micelles consisted only of poloxamers. In an emulgel with an emulsifier (EPG), the micelles consist of a poloxamer and polysorbate 80, so the structure of the micelles is maintained and the formulation remains unchanged even at −20 °C.

### 3.7. In Vitro Release Test

In vitro release studies are an important step in the development of pharmaceutical products. The chosen formulation and excipients must ensure adequate release of the active substance in order for the product to have adequate efficacy.

**Figure 7:**
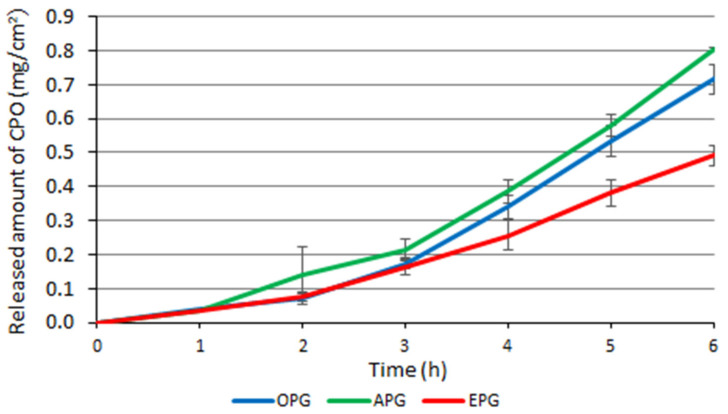
The profiles of ciclopirox olamine release from experimental semi-solid systems. At least three replicates were evaluated for each value. The error bars represent the standard deviation of the respective textural data.

An in vitro release assay was performed to determine the release of ciclopirox olamine over 6 h from different formulations. The fluxes of ciclopirox olamine from the OPG, APG, and EPG formulations within 6 h were 0.716 (0.006) mg/cm^2^, 0.805 (0.042) mg/cm^2^, 0.491 (0.029) mg/cm^2^, respectively (Figure 7). Ciclopirox olamine was released from the EPG at statistically significantly lower levels. An emulsifier (polysorbate 80) was used in the EPG formulation to help dissolve the ciclopirox olamine in the formulation. No statistically significant difference was found between the APG and OPG formulations, so the different viscosity of the formulations did not affect the release of the drug.

The micellar system is stable in the emulgel and ciclopirox olamine is a relatively lipophilic substance, so it remains in the micellar system and therefore its release from the EPG formulation is lower. Meanwhile, emulgel APG and bigel OPG form a weaker micellar system, resulting in the higher release of ciclopirox.

Due to the cellulose membrane and diffusion medium (purified water) used, back diffusion is possible in this test. This decreases the viscosity of the formulations and may lead to a better release of the active substance. The tested formulations contained the same amount of hydrogel, so this effect should be the same for all formulations tested.

The study found that ciclopirox was slowly released from the tested emulgels and bigel. Other researchers have found prolonged release of the following active substances from emulgels: ibuprofen [20], ciclopirox olamine [30], and vancomycin [19]. This release profile is appropriate for antifungal formulations when the drug is used for a long time and the presence of the drug at the site of action must be ensured [35].

The release of active substances depends on the properties of the active substance; therefore, studies with other active substances are planned in the future to evaluate how the addition of additional excipients (emulsifiers, gelling agents) changes the release and penetration of active substances into human skin.

### 3.8. Antifungal Activity Study

To determine whether the composition of the formulations affects activity, antifungal studies were performed with the *T. rubrum*. Emulgels and bigel with ciclopirox olamine produced inhibition zone diameters of 45 to 52 mm for all the isolates tested in the study (Table 6). The study showed that the diameter of the inhibition zone of the EPG formulation is slightly smaller than of OPG and APG. This may be related to the lower release of ciclopirox olamine from the EPG formulation. However, the difference found is not statistically significant and all formulations tested are effective against *T. rubrum*. Formulations without the pharmaceutical substance showed no inhibition.

The data on ciclopirox effectivity against dermatophytes agree with the results presented in our [36] and other scientists’ recent studies [37,38].

## 4. Conclusions

Studies have shown that biphasic systems with poloxamer gel can be produced in a variety of ways: oil is added pure, oil is added with emulsifiers, or oil is gelled. The particle size of all dispersion systems tested was in the range 0.214–2.13. Gelation of the oil phase (OPG) provides stronger mechanical properties (firmness, consistency, index of viscosity). Of all formulations tested, EPG maintained the gel structure at 10 °C. All investigated formulations remained stable during the centrifugation and cooling–heating cycles, and only the emulgel with polysorbate 80 (EPG) withstood the freeze–thaw cycles and remained stable. The release of ciclopirox olamine from the emulsion with polysorbate 80 was slower than from the other formulations tested, but their antimicrobial activity was similar. In order to produce a thermally stable semi-solid emulsion system, it is advisable to add an emulsifier while mixing the poloxamer gel with the oil.

Emulgels with a poloxamer are a scientifically interesting pharmaceutical form. In the future, it is planned to continue research and evaluate the influence of different emulsifiers and their concentrations, different oils on the stability and properties of emulgels with a poloxamer. Emulgels with different active ingredients will also be produced to evaluate the effect of the oil incorporation method on the release of different sizes and lipophilically active substances. The penetration of active substances into human skin will also be assessed.

## Figures and Tables

**Table 1:** Compositions of the experimental semi-solid formulations.

Components	APG (%)	EPG (%)	OPG (%)
Purified water	36.5	36.5	36.5
Poloxamer 407	12.5	12.5	12.5
Mineral oil (light)	50.0	47.5	47.5
Silicon dioxide	–	–	2.5
Polysorbate 80	–	2.5	–
Ciclopirox olamine	1	1	1

**Table 2:** Percentile (D10) values after production and after 1, 2, 3, and 4 weeks. The results are presented as specified mean (standard deviation).

	OPG (µm)	APG (µm)	EPG (µm)
0 week	0.337 (0.051)	0.327 (0.027)	0.286 (0.008)
1 week	0.285 (0.025)	0.330 (0.014)	0.275 (0.016)
2 weeks	0.284 (0.028)	0.337 (0.020)	0.287 (0.009)
3 weeks	0.284 (0.026)	0.338 (0.028)	0.290 (0.010)
4 weeks	0.283 (0.023)	0.371 (0.009)	0.297 (0.021)

**Table 3:** Percentile (D50) values after production and after 1, 2, 3, and 4 weeks. The results are presented as specified mean (standard deviation).

	OPG (µm)	APG (µm)	EPG (µm)
0 week	0.420 (0.046)	0.484 (0.055)	0.470 (0.085)
1 week	0.421 (0.034)	0.495 (0.029)	0.404 (0.036)
2 weeks	0.422 (0.039)	0.508 (0.039)	0.427 (0.012)
3 weeks	0.422 (0.036)	0.510 (0.048)	0.433 (0.014)
4 weeks	0.422 (0.033)	0.567 (0.015)	0.442 (0.030)

**Table 4:** Percentile (D90) values after production and after 1, 2, 3, and 4 weeks. The results are presented as specified mean (standard deviation).

	OPG (µm)	APG (µm)	EPG (µm)
0 week	0.700 (0.077)	0.865 (0.102)	0.716 (0.021)
1 week	0.705 (0.058)	0.869 (0.080)	0.681 (0.064)
2 weeks	0.705 (0.064)	0.893 (0.094)	0.725 (0.02)
3 weeks	0.707 (0.058)	0.890 (0.101)	0.732 (0.019)
4 weeks	0.708 (0.055)	0.992 (0.026)	0.744 (0.037)

**Table 5:** Results of emulgels and bigel texture properties.

	OPG	APG	EPG
Firmness (g)	2213.31 (46.16)	1562.08 (91.21)	1763.37 (134.02)
Consistency (g·s)	8257.48 (492.92)	6024.86 (348.29)	6653.80 (741.92)
Cohesiveness (g)	−1983.57 (60.44)	−1414.63 (76.24)	−1623.47 (142.97)
Index of viscosity (g·s)	−6793.20 (868.51)	−5255.84 (237.74)	−5526.72 (257.89)

**Table 6:** Inhibition zone diameter.

	OPG (mm)	APG (mm)	EPG (mm)
Inhibition zone diameter	49.8 (0.84)	49.8 (1.48)	47.2 (1.48)

## Data Availability

Data available in a publicly accessible repository.
